# The ability to race barefoot is a heritable trait in Standardbred and Coldblooded trotters

**DOI:** 10.1186/s12711-025-00958-2

**Published:** 2025-02-25

**Authors:** Paulina Berglund, Sreten Andonov, Anna Jansson, Christina Olsson, Therese Lundqvist, Erling Strandberg, Susanne Eriksson

**Affiliations:** 1 Department of Animal Biosciences, Swedish University of Agricultural Sciences, P.O. Box 7023, 75007 Uppsala, Sweden; 2 Swedish Trotting Association, P.O. Box 20151, 16102 Bromma, Sweden

## Abstract

**Background:**

In equine sports, shoes are used to protect the hooves from wear and tear. In Swedish trotting races, pulling off the shoes to race barefoot is popular because it improves racing time. Good hoof quality is essential for high-performance horses, but not all trotting horses have hooves that tolerate barefoot racing. The ability to race barefoot is a complex trait that is known to be influenced by environmental factors, but the genetic basis of this trait has not been studied. The aim of this study was to estimate genetic parameters and correlations between estimated breeding values for three novel traits: two related to the proportion of barefoot races and “barefoot status”, a binary trait that reflects the probability of racing unshod in a race, in Swedish Standardbred trotters (SB) and Swedish-Norwegian Coldblooded trotters (CB).

**Results:**

For the two traits describing the proportion of barefoot races, single-trait mixed linear animal models were used to estimate variance components for up to 24,958 SB and up to 4050 CB. Estimates of heritability ranged from 0.17 to 0.28. For barefoot status, a binary trait with repeated measurements, 875,056 observations from 25,973 SB, and 93,376 observations from 3384 CB were included. Using a single-trait mixed animal threshold model estimates of heritability for barefoot status were 0.07 and 0.08. The Pearson correlation coefficient between the estimated breeding values for barefoot status and each of the traits describing the proportion of barefoot races for breeding stallions was 0.63 and 0.64 for SB and 0.82 and 0.76 for CB.

**Conclusions:**

The traits analyzed reflecting the ability to race barefoot are heritable, with the traits for the proportion of barefoot races showing higher heritability estimates for both breeds than barefoot status. Estimated breeding values for breeding stallions were moderately to strongly correlated for the three traits. The average accuracy of estimated breeding values for breeding stallions was moderate to high for all traits. To breed for the ability to race barefoot, further studies on the genetic correlation of the ability to race barefoot with performance traits and the impact of racing barefoot on career length, are necessary.

## Background

“No hoof, no horse” is a well-known saying that pinpoints the importance of good hoof quality for health and performance of horses. In Swedish trotting races, pulling off the shoes to compete barefoot is a common practice to increase the speed of the horse [1]. Racing fully barefoot has been shown to reduce racing time on average by 0.7 s per km in Swedish Standardbred trotters, but also to increase the risk of disqualification and breaking over to gallop [1]. Racing with barefoot hind hooves and breaking over to gallop can increase the risk of injuries to the front limbs as a result of interference (i.e. overreaching, brushing, and forging) [2]. However, these results are inconclusive, as a study on Italian Standardbred trotters did not show a significantly elevated risk of retrieving musculoskeletal injuries from racing unshod [3].

Since the end of 2004, information about Swedish trotting horses’ shoeing conditions at competitions has been recorded, which is part of the information that bettors receive before the race due to its impact on performance. The Swedish Trotting Association has special regulations regarding barefoot racing, with barefoot racing not being allowed during the winter season nor with 2-year-old horses [4].

There seems to be variation in the ability to race barefoot among Swedish trotters and not all horses have hooves that can tolerate barefoot racing. Previous studies have suggested that the hind hooves are the limiting factor for whether a horse can endure barefoot racing or not [1, 5]. Hence, trainers often choose for the horse to race with barefoot front hooves in combination with shod hind hooves, to increase the speed without elevating the risk of disqualification [1]. Horses that gallop repeatedly, gain position in gallop, gallop a certain distance, or cross the finishing line in gallop are disqualified [4]. Approximately one-third of all starts in Swedish races for 4- to 15-year-old Standardbred trotters were made with fully barefoot horses [6]. The corresponding fraction for fully barefoot Coldblooded trotter starts was one-fourth [6]. Nevertheless, the most common shoeing condition in Swedish trotting races is fully shod. Racing with shoes can also help to balance the gait, especially for Coldblooded trotters, which need the weight of the shoes to trot correctly to a greater extent than Standardbred trotters [6]. It is also possible to race with barefoot hind hooves and shod front hooves, but this is less common [1]*.*

Despite its impact on performance, the genetic background of the ability to race barefoot has not been studied. Traits related to hoof conformation have been shown to be important for performance for several equine sports [7–10] and for longevity for sport horses [7]. Genetic studies on hoof conformation traits have shown estimates of heritability ranging from 0.02 to 0.52 for trotting horses of different breeds: Coldblooded trotter, Finnhorse, and Standardbred trotter [8, 11–13].

The objective of this study was to investigate whether it is possible to breed for the ability to race barefoot as an indirect measurement of hoof quality in Swedish Standardbred trotters and Swedish-Norwegian Coldblooded trotters. The study aimed at estimating genetic parameters and correlations between estimated breeding values for three novel traits. The first two traits, were related to the ability to race repeatedly with barefoot hind hooves measured as the proportion of barefoot races, and the second trait, “barefoot status”, was a binary trait with repeated observations of whether horses started in a race with bare hind hooves or not. Knowledge about the heritability of the ability to race barefoot could serve as a tool to improve the breeding programs for the Swedish Standardbred trotter and the Swedish-Norwegian Coldblooded trotter with regard to performance, health, and welfare.

## Methods

### Data and pedigree

Data on Swedish trotting races and pedigree information for Swedish Standardbred trotters (SB) and Swedish-Norwegian Coldblooded trotters (CB) were provided by the Swedish Trotting Association. Shoeing condition during the race, i.e., whether the horse was competing with shod front hooves, shod hind hooves, fully shod, or fully barefoot, have been recorded since the end of 2004 for trotting races in Sweden. The original dataset for SB consisted of 1,442,500 racing records from 64,350 horses that competed from 2005 to 2022. The CB race data included performance data from 2005 to 2021 for 173,141 racing records from 8685 horses before data editing. The original pedigree file for SB contained 305,820 animals and that for CB included 118,239 animals.

### Trait definition and data editing

From the available information, three trait definitions were formed and used for analysis: two traits related to the proportion of barefoot races and barefoot status. For these traits, barefoot was defined as racing with barefoot hind hooves, but in most cases, the horses were fully barefoot.

#### Proportion of barefoot races

The proportion of barefoot races was defined as the relative frequency of races in which the horse was barefoot on the hind hooves and shod or barefoot on the front hooves. Two separate traits (I and II) were defined for the proportion of barefoot races. For both traits, the horses were required to have started in at least 10 races for SB and 5 for CB. For the trait proportion of barefoot races II, there was an added requirement of the horse having at least one race with barefoot hind hooves, which removed 3647 non-barefoot racers for SB and 2130 for CB.

Observations were removed for both traits if they were from monté races (i.e., ridden races), had missing shoeing information, had missing pedigree, or if the horse was born outside of Sweden (only for SB). Only observations from March 1st to November 30th were included because racing barefoot in the winter (December to February) has not been allowed since December 1, 2015. Observations recorded for the track condition “winter track” that occurred outside of the winter season were also removed because no barefoot observations were registered for this track condition. Only racing results from SB born from 2002 to 2018 that were 3 to 10 years of age at the time of the race were included in the analysis. The CB horses included in the study were born between 2002 and 2017 and the results were kept from races in which they were 3 to 10 years old.

The number of observations that remained in the edited dataset used for analyses for the proportion of barefoot races I and II is shown in Table 1. In both breeds, more than half (55 and 64%) of the horses with observations for these traits were geldings or stallions (Table 2). The number of SB and CB horses per birth year was at least 818 and 61, respectively.

**Table 1:** Numbers of observations and number of horses (in parentheses) in the original dataset and for each trait after editing for Standardbred (SB) and Swedish-Norwegian Coldblooded (CB) trotters

Trait/dataset	Number of observations (horses)	
	SB	CB
Original dataset	1,442,500 (64,350)	173,141 (8685)
Prop. barefoot races I	724,232 (24,928)	97,682 (4050)
Prop. barefoot races II	649,091 (21,281)	60,292 (1947)
Barefoot status	875,056 (25,973)	93,376 (3384)

**Table 2:** Least squares (LS) means and standard errors (SE) for the proportion of barefoot races I and II for Swedish Standardbred trotters (SB) and Swedish-Norwegian Coldblooded trotters (CB)

Breed	Trait	Factor	Levels	Number of horses	LS means	SE
SB	Prop. Barefoot races I	Birth year	2002	1573	0.27	0.007
			2018	1023	0.31	0.008
		Sex	Mare	11,151	0.29	0.003
			Gelding/stallion	13,777	0.30	0.002
	Prop. Barefoot races II	Birth year	2002	1294	0.28	0.006
			2018	813	0.35	0.009
		Sex	Mare	9463	0.30	0.003
			Gelding/stallion	11,818	0.30	0.002
CB	Prop. Barefoot races I	Birth year	2002	244	0.08	0.011
			2017	170	0.09	0.013
		Sex	Mare	1629	0.08	0.004
			Gelding/stallion	2421	0.11	0.003
	Prop. Barefoot races II	Birth year	2002	112	0.12	0.010
			2017	61	0.19	0.024
		Sex	Mare	692	0.12	0.005
			Gelding/stallion	1255	0.13	0.004

The LS means from birth years 2003–2017 for SB and from 2003–2016 for CB are not included in the table

The main reduction in observations in both traits for both breeds was due to the removal of observations from monté races (46,119 for SB and 4597 for CB), foreign horses (139,170 for SB only), and winter races (races in December, January and February, 181,669 for SB and 24,082 for CB), but the order of the editing rules applied as described above may have influenced this.

#### Barefoot status

The trait barefoot status was created as a binary trait with repeated observations, where each race for a horse was coded as 2 if it had raced with barefoot hind hooves and 1 otherwise. Observations were removed based on the same criteria as for the proportion of barefoot races I and II, except that data for races in the winter were kept if the track condition was not winter track (only races until 2015). In addition, observations for barefoot status were removed if they were from the starting method “Line start” (not standard, only used when the starting car was not working and therefore few observations had this starting method), or if they were from tracks with few observations. Seven tracks were removed for SB and six for CB, resulting in 33 tracks remaining for SB and 24 for CB. The number of observations per track ranged from 1422 to 105,060 for SB and from 141 to 12,334 for CB.

The SB horses were required to be born between 2002 and 2018 to be included and CB horses between 2002 and 2017. For both breeds, data from races when they were 3 to 10 years old were kept.

Data from trainers without a barefoot race in the dataset were removed to avoid the influence of trainers who were opposed to barefoot racing, which excluded 27% of the trainers of SB horses and removed 3% of the data. In total, 61% of the CB trainers were excluded, which eliminated 18% of the data. Finally, SB horses were required to have started at least 10 races, and CB at least five races to be kept. For this trait, no requirement for the number of barefoot races per horse was made.

The total numbers of observations and number of horses included in the final dataset for barefoot status are shown in Table 1. For SB, 28% of the races were made with barefoot hind hooves and of these, 74% were made fully barefoot. For CB, 11% of the races were with barefoot hind hooves and of these, 59% were made fully barefoot. The use of repeated observations of barefoot status in multiple races allowed for adjustment for race-specific fixed effects in the statistical model, as further described below.

#### Definition of fixed effects

Each race included information about the date, track, track condition, starting method, race distance, prize money, trainer, and trainer level. Because distribution of races across the country differs for the two breeds, with CB mainly competing in the northern part of Sweden, season of racing was defined differently for the two breeds. For SB, the seasons were defined as winter between December 1 and February 28, spring between March 1 and May 11, early summer between May 12 and July 11, late summer between July 12 and September 31, and autumn between October 1 and November 31. Because the number of observations with barefoot hind hooves for CB during the winter season was small in the years when it was allowed (years 2005–2015 in the data), the winter season was split into two and combined with autumn and spring observations, respectively. As a result, for CB, winter-spring was from January 16 to May 24, early summer from May 25 to July 11, late summer from July 12 to September 24, and autumn-winter from September 25 to January 15 for the CB.

For each race, the track condition was noted as an indicator of the hardness of the track, classified as easy, somewhat heavy, or heavy. For CB, observations with heavy and somewhat heavy track conditions were combined due to relatively few (1155) observations for the former. Observations from winter track conditions were not included for either breed, although observations from other track conditions during the winter season prior to 2015 were included (from 2015 and onwards, racing barefoot was not allowed in winter).

There were two types of starting methods: auto-start (start behind a car) and volt-start (circle start with a maximum of 5 horses per circle). Races were divided into three race distances [14] according to the Swedish Trotting Association’s definitions: short-distance (1640 m), medium-distance (2140 m), and long-distance (2640 m). A fourth race distance named marathon (up to 4140 m) was added to account for the 15,432 observations for SB from races that exceeded the distance of the long-distance races.

The level of prize money for the winner in a race, reflecting the level of the race, was grouped into 14 classes for SB and nine classes for CB. The number of observations per class ranged from 1164 to 137,081 for SB and from 1044 to 18,061 for CB.

Trainers were classified as professionals or amateurs based on current definitions by the Swedish Trotting Association. Trainers with type A license were classified as professionals, and trainers with type B license were classified as amateurs. In total, there were 4997 unique trainers of SB and 1017 unique trainers of CB.

### Statistical models

#### Proportion of barefoot races

**Fig. 1:**
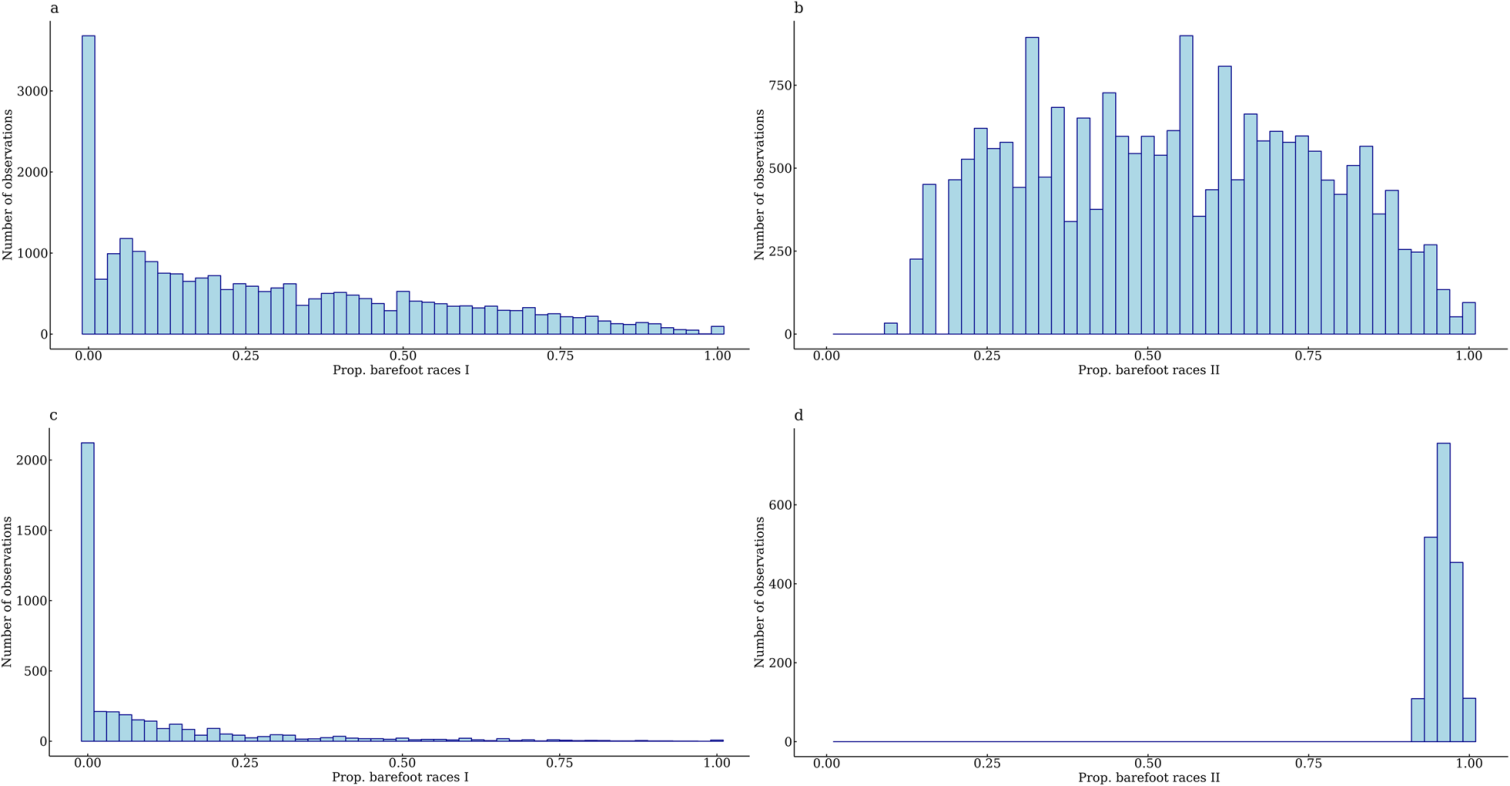
Trait distribution for the proportion of barefoot races I and II. For Swedish Standardbred trotters, **a** shows the trait distribution for proportion of barefoot races I and **b** shows the trait distribution for proportion of barefoot races II after square root transformation. In **c**, the distribution of proportion of barefoot races I in Swedish-Norwegian Coldblooded trotters is shown and in **d** the distribution of proportion of barefoot races II is shown after Box-Cox transformation with λ = 0.02

A linear model with the proportion of barefoot races as the response variable and sex and year of birth as fixed effects was analyzed with the function lm in RStudio [15] to estimate least squares means. Because of the combined trait information from all included races for each horse, only fixed effects that were constant across races could be adjusted for in the statistical model (described below). The proportion of barefoot races I was not transformed because it did not help making the distribution of residuals normal. The proportion of barefoot races II was transformed to make the residuals normally distributed. The appropriate power transformation was determined using the Box-Cox function from the MASS package [16] in R [15], separately for each breed, resulting in a square root transformation for SB and raising to the power 0.02 for CB. The trait distributions for proportion of barefoot races I and II (after transformation) are shown in Fig. 1a and b for SB and in Fig. 1c and d for CB.

Variance components were estimated separately for each breed with a single-trait mixed linear animal model with AIREML of the BLUPF90+ (version 2.47) family of programs [17], followed by a separate run of BLUP with the same package to estimate breeding values, using the following model:

1 $${\mathbf{y}} = {\mathbf{Xb}} + {\mathbf{Za}} + {\mathbf{e}},$$

where **y** is the proportion of barefoot races (I or II), **X** and **Z** are incidence matrices, **b** is the vector of two fixed effects: sex (mare or gelding/stallion) and year of birth (2002–2018 for SB and 2002–2017 for CB), **a** is the vector of additive genetic effects ~ N(**0**,**A** ${\upsigma }_{\text{a}}^{2}$), where** A** is the numerator relationship matrix, and ${\upsigma }_{\text{a}}^{2}$ is the additive genetic variance, and **e** is the vector of residuals ~ N(**0**,$\mathbf{I}{\upsigma }_{\text{e}}^{2}$), where **I** is the identity matrix and ${\upsigma }_{\text{e}}^{2}$ the residual variance. A pedigree of seven generations with 51,675 SB and 8056 CB horses was used to derive **A** for proportion of barefoot races I. For proportion of barefoot races II, the corresponding numbers were 56,968 for SB and 11,870 for CB. Diagonal elements of **A** accounted for inbreeding.

#### Barefoot status

Preliminary mixed generalized linear models for the binary trait barefoot status were analyzed with PROC GLIMMIX in SAS [18] to decide on fixed effects and definitions of the classes. The final model in SAS included the random effect of horse and the fixed effects of age, sex, year, season, track, track condition, starting method, race distance, prize money, and trainer level. Level of significance, least squares means, and inverse link transformations (predicted probability) of least squares means were estimated for each fixed effect. Only the random effect of the horse was included in the preliminary model because additional random effects (trainer and year-season interaction) caused problems in SAS due to insufficient memory.

Variance components and breeding values were estimated using the GIBBSF90+ (version 3.16) program of the BLUPF90 software [17]. The following single-trait animal threshold model with repeated observations was used:

2 $${\text{y}} = {\mathbf{Xb}} + {\mathbf{Z}}_{{\text{s}}} {\mathbf{s}} + { }{\mathbf{Z}}_{{\text{t}}} {\mathbf{t}} + { }{\mathbf{Z}}_{{\text{a}}} {\mathbf{a}} + { }{\mathbf{Z}}_{{\text{p}}} {\mathbf{p}} + {\mathbf{e}},$$

where **y** is the vector of the binary trait shod or barefoot, **X**, **Z**_**s**_, **Z**_t_,** Z**_a,_ and** Z**_p_ are incidence matrices, **b** is a vector of fixed effects, vector **s** is the random effect of the year-season interaction ~ N(**0**,**I** ${\upsigma }_{\text{s}}^{2}$), where **I** is the identity matrix, vector **t** is the random effect of the trainer of the horse ~ N(**0**,**I** ${\upsigma }_{\text{t}}^{2}$), **a** is the vector of additive genetic effects ~ N(**0**,**A** ${\upsigma }_{\text{a}}^{2}$), where** A** is the numerator relationship matrix, where ${\upsigma }_{\text{a}}^{2}$ is the additive genetic variance, vector **p** is the random permanent environmental effect ~ N(**0**,**I** ${\upsigma }_{\text{p}}^{2}$), and **e** is the vector of residuals ~ N(**0**,**I** ${\upsigma }_{\text{e}}^{2}$), where ${\upsigma }_{\text{e}}^{2}$ is the residual variance, which was set to 1. The **A** matrix was constructed based on seven generations of pedigree with 58,070 animals for SB and 10,464 for CB and with the diagonal element accounting for inbreeding. Vector **b** included 10 fixed effects: starting method (volt-start or auto-start), track (33 levels for SB and 24 levels for CB), track condition (easy, somewhat heavy or heavy for SB and easy or somewhat heavy/heavy for CB), sex (mare or gelding/stallion), age (3–10 years), race year (2005–2022 for SB and 2005–2021 for CB), season (winter, spring, early summer, late summer, autumn or winter for SB; and winter-spring, early summer, late summer or autumn-winter for CB), race level (14 levels for SB and nine for CB), trainer level (professional or amateur) and distance (short-, medium-, long- or (for SB) marathon-distance).

Multiple Gibbs sampling runs with different chain lengths, burn-ins, and thinnings were tested and passed to post-Gibbs analysis. The appropriate chain lengths were decided by analyzing the size of the effective samples and the thinning was based on the sample’s autocorrelation. After post-Gibbs analyses and visual inspection of the chains in RStudio to check if they had reached convergence, the final options for SB were set to 400,000 iterations, 60,000 as the burn-in, and a thinning of 80. For CB, the options were set to 700,000 iterations, 80,000 burn-in, and a thinning of 80. In total, 4250 samples for SB and 7750 samples for CB were passed to post-Gibbs analysis in POSTGIBBSF90.

#### Estimation of heritability, repeatability, and accuracy of estimated breeding values

The heritabilities (${\text{h}}^{2}$) of the two proportions of barefoot races traits were estimated based on estimates of variance components (^) as:

$${\hat{\text{h}}}^{2} = { }\frac{{{\hat{\upsigma }}_{{\text{a}}}^{2} }}{{{\hat{\upsigma }}_{{\text{a}}}^{2} + {\hat{\upsigma }}_{{\text{e}}}^{2} { }}}.$$

The heritability of barefoot status was estimated as the posterior mean of heritability obtained from the sampled variances at each iteration (^) as:

$${\hat{\text{h}}}^{2} = { }\frac{{{\hat{\upsigma }}_{{\text{a}}}^{2} }}{{{\hat{\upsigma }}_{{\text{a}}}^{2} { } + {\hat{\upsigma }}_{{\text{p}}}^{2} + {\hat{\upsigma }}_{{\text{e}}}^{2} { }}}.$$

Repeatability for barefoot status was estimated from the posterior means of variance components (^) as:

$${\text{r}} = { }\frac{{{\hat{\upsigma }}_{{\text{a}}}^{2} + {\hat{\upsigma }}_{{\text{p}}}^{2} }}{{{\hat{\upsigma }}_{{\text{a}}}^{2} { } + {\hat{\upsigma }}_{{\text{p}}}^{2} { } + {\hat{\upsigma }}_{{\text{e}}}^{2} { }}}.$$

For proportion of barefoot races I and II, the accuracy of the estimated breeding value of a horse (r_TI_) was based on the prediction error variance (PEV) derived from the mixed model equations by the BLUPF90 program and was calculated according to [19] as:

$${\text{r}}_{{{\text{TI}}}} = \sqrt {1 - \frac{{{\text{PEV}}}}{{\left( {1 + F_{i} } \right){\hat{\upsigma }}_{{\text{a}}}^{2} }}} ,$$

where ${F}_{i}$ is the individual’s inbreeding coefficient. For barefoot status, the accuracy was calculated manually using the same formula as above, using the squared posterior standard deviation provided by the GIBBSF90+ program as PEV for breeding values.

#### Correlations between estimated breeding values

Pearson and Spearman rank correlations between estimated breeding values (EBV) for the proportion of barefoot races and barefoot status were calculated for stallions born in 1992 and later that had at least 10 offspring with own racing performance in the data. Stallions that fulfilled these requirements for SB were born from 1992 to 2013, and those for CB were born from 1992 to 2011, resulting in the inclusion of 285 SB and 69 CB stallions for the proportion of barefoot races I, 270 for SB and 49 for CB for the proportion of barefoot races II, and 289 SB stallions and 60 CB stallions for barefoot status.

## Results

### Fixed effects and least squares means

#### Proportion of barefoot races

The raw overall mean values for the proportion of barefoot races I in the dataset after editing were 0.29 for SB and 0.10 for CB. For the proportion of barefoot races II, the corresponding numbers were 0.34 for SB and 0.20 for CB. In the mixed linear model fitted in RStudio [15], birth year was significant at p < 0.0001 for both traits in SB and at p = 0.88 and p = 0.07 for CB for proportion of barefoot races I and II, respectively. Least squares means for birth year, which were back-transformed to the original scale for the proportion of barefoot races II, showed an increased proportion of barefoot races for both trait definitions from 2002 to 2018 (SB) and from 2002 to 2017 (CB) (Table 2). However, the increase in barefoot races for CB over birth years was minor when horses that never raced barefoot were included (proportion of barefoot races I) (from 8% for horses born in 2002 to 9% for horses born in 2017). Sex was not significant at p = 0.12 for SB but significant at p < 0.0001 for CB for the proportion of barefoot races I. Corresponding p-values for the proportion of barefoot races II were 0.91 and 0.02, respectively. Least squares means for sex, back-transformed to the original scale for the proportion of barefoot races II, were similar for SB geldings/stallions and mares for both traits (Table 2). For CB, geldings/stallions had a somewhat higher proportion of barefoot races than mares when including horses that never had raced barefoot (proportion of barefoot races I).

#### Barefoot status

Least squares means, expressed as the probability of racing barefoot for the fixed effects starting method, track condition, sex, trainer level, and distance group for SB are in Table 3. All fixed effects included in the model were significant at p < 0.0001. The probability of racing barefoot was higher in races in which the starting method was auto-start, on easy track conditions, and in short races. If the horse was trained by a professional trainer or was a mare, the probability of racing barefoot was higher in comparison to horses trained by an amateur trainer or if the horse was a gelding/stallion.

**Table 3:** Least squares (LS) means and standard errors (SE) for barefoot status for levels of fixed factors in Swedish Standardbred trotters

Factor	Level	Number of horses	Number of observations	LS means	SE
Starting method	Auto-start	25,750	493,143	0.212	0.005
	Volt-start	25,792	381,913	0.173	0.004
Track condition	Easy	25,973	822,070	0.239	0.003
	Somewhat heavy	19,314	50,713	0.187	0.004
	Heavy	2000	2273	0.156	0.010
Sex	Mare	11,713	360,085	0.200	0.005
	Gelding/stallion	14,260	514,971	0.184	0.005
Trainer level	Professional	20,264	513,562	0.248	0.006
	Amateur	14,993	361,494	0.146	0.004
Distance group	Short	22,866	155,870	0.222	0.005
	Medium	25,971	619,172	0.188	0.005
	Long	18,141	84,582	0.187	0.005
	Marathon	5004	15,432	0.173	0.005

LS means are expressed as a probability of racing barefoot. The fixed effects track, age, season, prize money and year are not included in the table

**Fig. 2:**
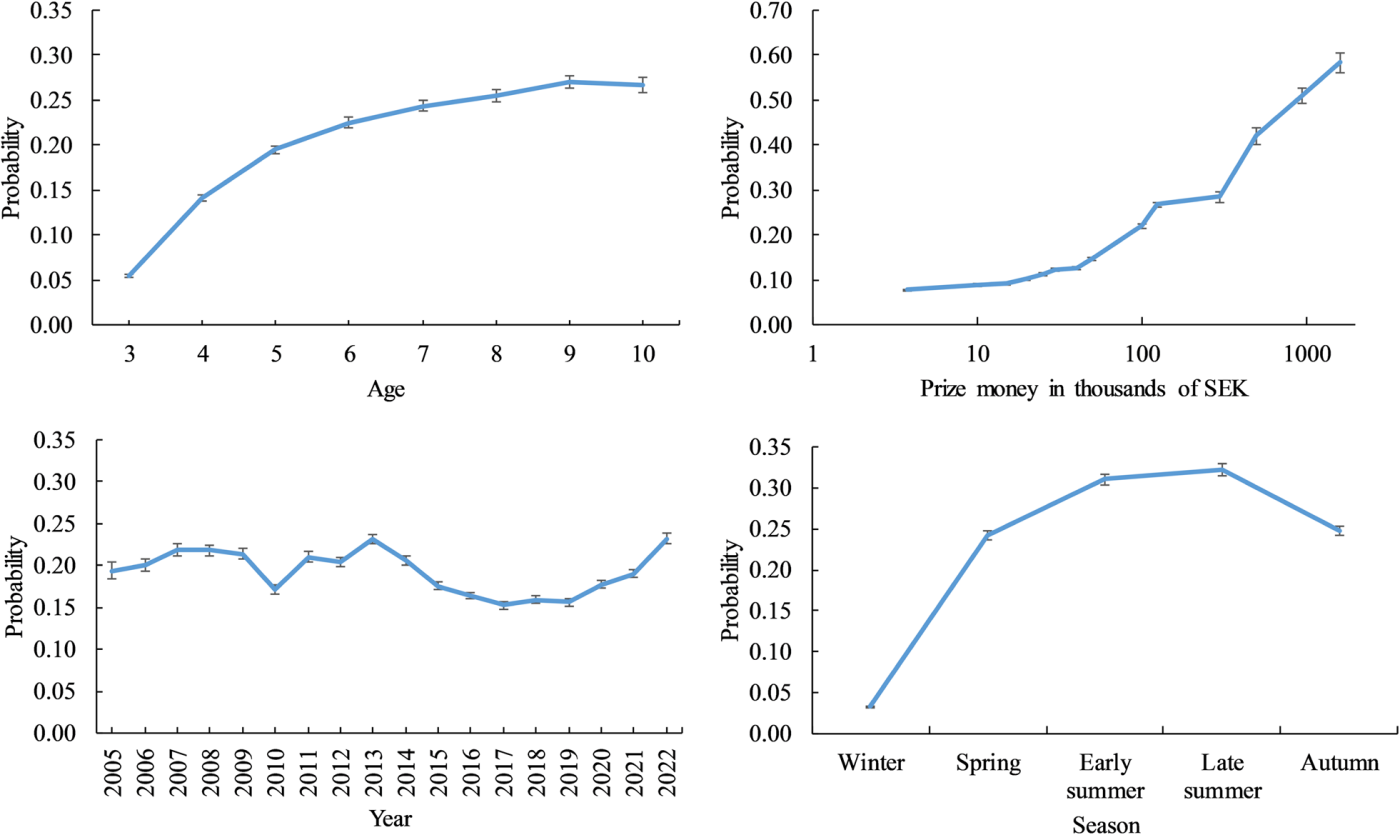
Least squares mean (probability) of racing barefoot for the trait barefoot status. The probability for each level of the fixed effects is shown: age (years), prize money (where the median price money for each class represents the points on the x-axis in thousands of SEK), year and season in Swedish Standardbred trotters. Bars represent standard errors

In Fig. 2, least squares means (probability) of racing barefoot are shown for age, prize money, year, and season. The probability of racing barefoot was 0.055 and 0.267 for 3- and 10-year-old SB horses, respectively. Prize money had a large effect on whether the horse raced barefoot or not. For races with prize money < 5 K SEK (median 3.75 K SEK), the probability of racing barefoot was 0.077 ± 0.002, compared with 0.583 ± 0.022 for races with prize money > 1 M SEK (median 1.6 M SEK). The probability of racing barefoot has been relatively stable over birth years, fluctuating between 0.15 and 0.23. After 2015, when new regulations for winter races were put in place, the probability of racing barefoot decreased. However, since 2019, the probability of racing barefoot has increased again. Seasonal changes also played an important role in the probability of racing barefoot in SB trotters, with late summer having the highest probability, followed by early summer.

Least squares means (probability) of racing barefoot for CB are shown in Table 4. All fixed effects included in the model were significant at p < 0.0001 except for sex (p = 0.04) and distance (p = 0.001). As for SB horses, the probability of racing barefoot for CB horses was higher for races with starting method auto-start, on easy track conditions, and in short-distance races. Also, CB horses trained by professional trainers had a higher probability of racing barefoot than horses trained by amateurs.

**Table 4:** Least squares (LS) means and standard errors (SE) for barefoot status for levels of fixed factors in Swedish-Norwegian Coldblooded trotters

Factor	Level	Number of horses	Number of observations	LS means	SE
Starting method	Auto-start	2592	17,537	0.071	0.004
	Volt-start	3377	75,839	0.062	0.003
Track condition	Easy	3384	85,475	0.079	0.004
	Somewhat heavy	2511	7901	0.056	0.004
Sex	Mare	1303	32,978	0.063	0.004
	Gelding/stallion	2081	60,398	0.071	0.004
Trainer level	Professional	2123	42,766	0.073	0.004
	Amateur	2052	50,610	0.061	0.004
Distance	Short	3119	21,635	0.072	0.004
	Medium	3378	64,257	0.065	0.003
	Long	1730	7484	0.064	0.004

LS means are expressed as a probability of racing barefoot. Track, age, season, prize money and year are not included in the table

**Fig. 3:**
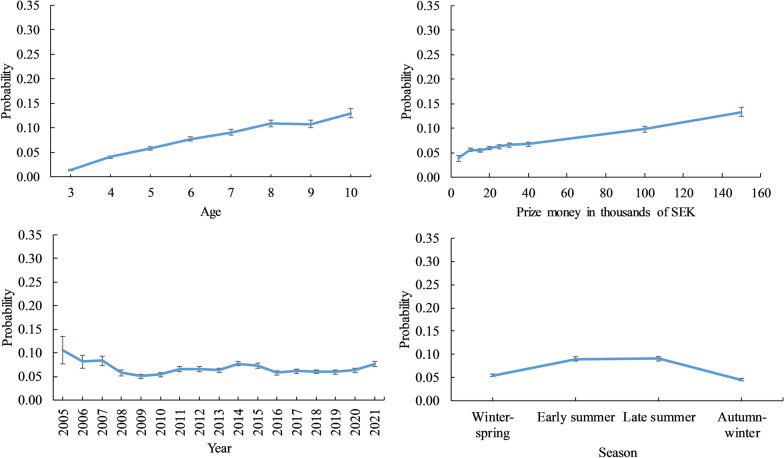
Least squares mean (probability) of racing barefoot for the trait barefoot status. The probability for each level of the fixed effects is shown: age (years), prize money (where the median price money for each class represents the points on the x-axis in thousands of SEK), year and season in Swedish-Norwegian Coldblooded trotters. Bars represent standard errors

The least squares means (probability) of racing barefoot for the fixed effects of age, prize money, year and season for CB horses are shown in Fig. 3. Similar to SB horses, CB horses were more likely to race barefoot at older ages and in races with more prize money. In 2005, the probability of racing barefoot peaked at 0.106 ± 0.028, while in 2009, the probability was as low as 0.052 ± 0.005. As for SB, the probability of racing barefoot was highest in the summer seasons.

### Variance components

Estimates of variance components, heritability, and repeatability for the evaluated traits are shown in Table 5. Estimates of heritability for proportion of barefoot races I and II were moderate: 0.28 ± 0.02, and 0.23 ± 0.02, respectively, in SB, and 0.17 ± 0.04 and 0.25 ± 0.06 in CB. For barefoot status, the estimate of heritability was 0.08 ± 0.01 for SB and 0.07 ± 0.03 for CB. Estimates of repeatability for barefoot status were moderate at 0.40 and 0.41 for SB and CB respectively.

**Table 5:** Estimates of variance components, heritability, and repeatability for the proportion of barefoot races I, II, and barefoot status for Standardbred trotters (SB) and Swedish-Norwegian Coldblooded trotters (CB)

Breed	Trait	${\upsigma }_{\text{s}}^{2}$	${\upsigma }_{\text{a}}^{2}$	${\upsigma }_{\text{t}}^{2}$	${\upsigma }_{\text{p}}^{2}$	${\upsigma }_{\text{e}}^{2}$	${\text{h}}^{2}$	r
SB	Prop. barefoot races I		0.020_0.002_			0.050_0.001_	0.284_0.020_	
	Prop. barefoot races II		0.011_0.001_			0.039_0.001_	0.225_0.020_	
	Barefoot status	0.830_0.155_	0.135_0.011_	0.316_0.010_	0.523_0.011_	1.000_0.002_	0.081_0.008_	0.397
CB	Prop. barefoot races I		0.005_0.001_			0.024_0.001_	0.171_0.038_	
	Prop. barefoot races II		0.0001_0.00003_			0.0003_0.00002_	0.254_0.064_	
	Barefoot status	0.007_0.003_	0.112_0.043_	0.266_0.024_	0.576 _0.040_	1.000_0.006_	0.066_0.026_	0.407

Posterior means for year season (${\upsigma }_{\text{s}}^{2}$), additive genetic (${\upsigma }_{\text{a}}^{2}$), trainer (${\upsigma }_{\text{t}}^{2}$), permanent environment (${\upsigma }_{\text{p}}^{2}$) and residual (${\upsigma }_{\text{e}}^{2}$) variance with heritability (${\text{h}}^{2}$) and repeatability (r) Standard deviations and posterior standard deviations are shown as subscript

### Accuracies and correlations between EBVs

For SB, the average accuracy of the EBVs was moderate to high (0.79–0.87) for breeding stallions born in 1992 and later for all studied traits (Table 6). The Pearson correlation coefficient between the EBVs of stallions for barefoot status and proportion of barefoot races I and II was 0.63 and 0.64, respectively. The corresponding Spearman rank correlation coefficient was 0.60.

**Table 6:** Mean and range of accuracies^a^ of estimated breeding values for the proportion of barefoot races and barefoot status for Swedish Standardbred (SB) and Swedish- Norwegian Coldblooded (CB) stallions with at least 10 progeny with data

Breed	Trait	Birth years	Number of stallions	Average number of offspring	Accuracy range	Mean accuracy
SB	Prop. barefoot races I	1992–2013	285	78.4	0.71–0.97	0.87
	Prop. barefoot races II	1992–2013	270	71.5	0.63–0.96	0.84
	Barefoot status	1992–2013	289	80.2	0.49–0.89	0.79
CB	Prop. barefoot races I	1992–2011	69	53.1	0.55–0.92	0.75
	Prop. barefoot races II	1992–2011	49	38.2	0.54–0.91	0.74
	Barefoot status	1992–2011	60	54.9	0.45–0.89	0.67

^a^ Accuracy (r_TI_) of the estimated breeding values (EBV) presented as range and mean for breeding stallions born in 1992 and later with at least ten offspring with data in Swedish Standardbred trotters (SB) and Swedish-Norwegian Coldblooded trotters (CB)

The average accuracy for the EBVs for the proportion of barefoot races and barefoot status ranged from 0.67–0.75 for CB breeding stallions born in 1992 and later (Table 6). The Pearson correlation coefficient between the EBVs for barefoot status and proportion of barefoot races I and II was 0.82 and 0.76, respectively. The corresponding Spearman rank correlation for CB of EBVs for barefoot status with those for the proportion of barefoot races I and II was 0.77 and 0.67, respectively.

## Discussion

We report the first genetic parameters for traits that reflect the ability of trotting horses to race barefoot. Having a well-functioning hoof is important for sports performance in horses [8–10]. Although information about shoeing conditions is routinely registered in Swedish trotting races, the potential of using this information in the breeding programs has not been studied. In this study, the trait proportion of barefoot races was created to reflect the ability to repeatedly race barefoot, and the binary trait barefoot status was created to reflect the probability of racing unshod in a race.

### Barefoot racing and hoof quality in horses

There is a general perception that hoof quality differs between breeds and between individual horses, indicating genetic variation, but scientific literature on this topic is scarce. In nonscientific articles, the Thoroughbred is perceived as a breed with poor hoof quality [20, 21], while Standardbred trotters are believed to have good hoof quality [22]. Standardbred trotters descend from Thoroughbreds but race on harder race tracks. Thus, it is reasonable to believe that Standardbreds have been indirectly selected for durable hooves. In a subpopulation of a Chinese-Mongolian horse breed adapted for life in the mountains and known for its strong and durable hooves, several candidate genes that could be important for hoof quality have been found [23], indicating genetic effects. In Swedish Standardbred trotters, individuals which are reported to have hooves that can tolerate racing barefoot repeatedly, have been found to have lower concentrations of copper and higher concentrations of arginine in the hoof wall compared with individuals that cannot race barefoot repeatedly, which could be linked to the hardness of the hoof [5]. In the present study, genetic parameters estimated for the proportion of barefoot races and barefoot status in Swedish trotters showed within-breed genetic variation for both traits for SB and CB.

### Trait definitions and environmental factors

The ability to race barefoot is a complex trait that is affected by the horse’s genes in combination with environmental factors and the trainer’s decisions. Due to the nature of the trait proportion of barefoot races, we could not adjust for the effect of the trainer. In our statistical analyses, we could only adjust for sex (mare or gelding/stallion) and year of birth, which were both the same for each horse across its racing years.

In the definition of the trait proportion of barefoot races I, we included horses that never raced barefoot. This trait has the advantage of covering a larger proportion of the population and the results are easier to interpret as no transformation was applied. For the trait proportion of barefoot races II, horses that had never started unshod hind were removed to help create a normally distributed trait as the proportion barefoot races I was zero-inflated, and to ensure that the included horses had the opportunity to race barefoot. In the end, both trait definitions gave similar results and, thus, the definition that includes more horses (I) is preferred.

The binary trait barefoot status defined in this study had the advantage of being based on repeated measurements, which made it possible to correct individual races for environmental effects of importance. This trait definition allowed more horses and observations to be retained for the analysis than for the traits based on the proportion of barefoot races. The trainer’s perception of barefoot racing could be an important factor in the decision to race barefoot or not. Therefore, observations from trainers who never raced with a barefoot horse in the data were removed to avoid that horses with the ability to race barefoot that never got the chance to do so, impact the results. The trainer of the trotting horse has been shown to explain more variation in racing performance than, for example, the driver of the horse [14]. Variation in the trait explained by the trainer may also cover yard-specific management routines such as feeding, housing, and farriery (hoof care). The trainer effect explained 11% and 14% of the phenotypic variance (defined as the sum of the variance components for all random effects in the model for genetic analysis) in SB and CB, respectively. However, the permanent environment effect for barefoot status was almost twice as large and represented 19% of the phenotypic variance for SB and 29% for CB. This effect accounts for factors not covered by the trainer effect, such as the horse’s history, including its rearing period, as well as for example previous training and injuries, but also variation that comes from trainers that only had one horse (more common in CB).

The amount of phenotypic variance explained by the random effect of year-season was 30% for SB but only 0.4% for CB. The probability of racing barefoot was relatively stable across years and seasons for CB compared with SB, for which the probability of racing barefoot varied more over years as well as over seasons.

There was a very small difference in the least squares mean for the proportion of barefoot races between sexes for both SB and CB. For barefoot status, the least squares mean from the preliminary analyses in SAS showed that SB mares had only a slightly higher probability of racing barefoot than SB geldings/stallions. This could be because mares are, on average, slower than geldings/stallions in trotting races [14], and removing the shoes could help to even out the differences in mixed races. A study on hoof strength and mineral content in different horse breeds reported no differences between sexes [24], which is in line with the findings of our study.

For barefoot status, easy track conditions resulted in a higher probability of racing unshod (SB and CB), followed by somewhat heavy (SB and CB) and heavy track conditions (SB). There was also a higher probability of racing unshod in short races than in longer races for both breeds. This could be due to smaller margins in shorter races that favor barefoot horses but also a potential increase of wear and tear of hooves for longer races.

The level of the race, which in this study was defined as the prize money for the winner, was shown to have a large impact on whether the horse raced barefoot or not. In SB races, prize money > 1 M SEK to the winner considerably increased the probability of racing barefoot, with a least squares mean as high as 60%. This shows the impact of prize money on the trainer taking the risk of racing barefoot. In CB races, for the class with the highest prize money to the winner the least squares mean for barefoot racing was only 13%. Possibly, the increased risk of disqualification associated with barefoot racing outweighs the benefit of increased speed when unshod.

### Heritability estimates

The estimated heritability for the proportion of barefoot races I and II was moderate (0.2–0.3) for both SB and CB. For these traits, the definition required at least 10 races for SB horses and 5 for CB horses, which may have influenced the estimate [25]. For barefoot status, the heritability estimate was low (< 0.1), although it was significant for both breeds and stable for different models and settings for Gibbs sampling and post-Gibbs analyses.

Variance component estimation for barefoot status with a threshold model required a relatively long computational time, especially for SB. However, the use of threshold models for a binary trait generally results in higher heritability than if it was treated as a linear trait [26, 27] because threshold models estimate the heritability on the underlying continuous scale. Threshold models also have the benefit of giving less biased heritability estimates for traits that are not normally distributed [28]. Despite the binary nature of barefoot status and the low heritability estimates, the large number of repeated observations led to moderate to high accuracy of EBV of breeding stallions, similar to the proportion of barefoot races.

### Hoof quality and racing performance

We considered the possibility that the proportion of barefoot races may be a too simplified measurement of the ability to race barefoot, as we could not take into account factors such as the level of the race, which appears to be of importance for the incentive to have the horse race barefoot. For barefoot status, factors such as prize money could be adjusted for, which would make this trait more independent from racing performance. The genetic correlation between the proportion of barefoot races and barefoot status could not be estimated because of convergence problems. However, the correlation between EBV for breeding stallions for the proportion traits and barefoot status trait was moderately strong and the corresponding genetic correlation can then be expected to be even higher. The simpler proportion traits thus seem to be rather closely related to the more strictly defined barefoot status trait, and thus useful as measures of the ability to race barefoot.

The difference in the popularity of racing barefoot for SB compared with CB horses may be due to reasons other than a difference in hoof quality. The low proportion of barefoot races for CB compared with SB horses is possibly partly due to genetic differences between the breeds in their ability to balance in trot at high speed. In Standardbred trotters, individuals that are homozygous for the A allele at the doublesex and mab-3 related transcription factor 3 (*DMRT3*) gene have been shown to be better at keeping trot at high speed and perform better in trotting races [29]. In the Coldblooded trotter, the benefit of the mutation in *DMRT3* on racing performance is less clear [30]. In Swedish Standardbred trotters, the favorable A allele is close to fixation, but in Coldblooded trotters the frequency of the A allele has been estimated to be 45% [31]. In Coldblooded trotters, often referred to as less good “natural trotters”, shoes still seem to play an important role in balancing the trot at high speed.

### Implications

This study presents the first estimates of the genetic contribution to the ability to race barefoot. Despite the low heritability for barefoot status for both breeds, it had the advantage of many repeated observations and that factors shown to be important for the trait, such as age, season, and prize money, could be accounted for. However, before implementing a trait that measures the ability to race barefoot in routine genetic evaluation, genetic correlation with performance traits must be estimated. Also, the welfare aspect of racing barefoot and its impact on the durability and longevity of trotting horses need to be studied, especially how shoeing condition in a horse’s early career impacts its future racing career. Today, European trotting associations have different regulations regarding barefoot racing for young horses [32] but there is a lack of published research to base new regulations and standards on.

Although the traits in this study were based on data that is already routinely recorded for both trotter breeds in Sweden, there are some practical concerns. During the part of the year when racing barefoot is allowed, the sole of the hoof can for instance be covered with plastic for protection and still be registered in the system as barefoot. If any of the traits studied in this paper were included in genetic evaluation of the two breeds, one would need to consider implementing a differentiation between barefoot and plastic covered soles in the registration. For the current study, information about this could have helped to verify that the information on shoeing condition was correctly interpreted.

## Conclusions

In this study, genetic parameters were estimated for the ability to race barefoot for Swedish Standardbred trotters and Swedish-Norwegian Coldblooded trotters, including two traits related to the proportion of barefoot races and the binary trait barefoot status. Heritability estimates ranged from 0.17 to 0.28 for the proportion of barefoot races I and II and from 0.07 to 0.08 for the barefoot status. Due to repeated observations for barefoot status, its average accuracy of EBV for breeding stallions was similar to that for the proportion of barefoot races traits. The Pearson correlation coefficient between the EBV of the proportion traits and barefoot status traits was 0.63 and 0.64 in SB and 0.82 and 0.76 in CB. These results indicate that it would be possible to select for the ability to race barefoot in trotters. However, further studies are needed to estimate the genetic correlation of these traits with performance and career length.

## Data Availability

The data used for this study were obtained from the Swedish Trotting Association and used under license. To access the data, permission is required from the Swedish Trotting Association.
